# Metabolomics-based profiling of 4 avocado varieties using HPLC–MS/MS and GC/MS and evaluation of their antidiabetic activity

**DOI:** 10.1038/s41598-022-08479-4

**Published:** 2022-03-23

**Authors:** Inas Y. Younis, Amira R. Khattab, Nabil M. Selim, Mansour Sobeh, Seham S. Elhawary, Mahitab H. El Bishbishy

**Affiliations:** 1 Pharmacognosy Department, Faculty of Pharmacy, Cairo University, Cairo, 11562 Egypt; 2 Pharmacognosy Department, College of Pharmacy, Arab Academy for Science, Technology and Maritime Transport, Alexandria, 1029 Egypt; 3 AgroBioSciences, Mohammed VI Polytechnic University, 43150 Ben-Guerir, Morocco; 4 Department of Pharmacognosy, Faculty of Pharmacy, MSA University, Giza, 12585 Egypt

**Keywords:** High-throughput screening, Drug screening, Target identification, Diseases

## Abstract

Seven avocado “*Persea americana*” seeds belonging to 4 varieties, collected from different localities across the world, were profiled using HPLC–MS/MS and GC/MS to explore the metabolic makeup variabilities and antidiabetic potential. For the first time, 51 metabolites were tentatively-identified via HPLC–MS/MS, belonging to different classes including flavonoids, biflavonoids, naphthodianthrones, dihydrochalcones, phloroglucinols and phenolic acids while 68 un-saponified and 26 saponified compounds were identified by GC/MS analysis. The primary metabolic variabilities existing among the different varieties were revealed via GC/MS-based metabolomics assisted by unsupervised pattern recognition methods. Fatty acid accumulations were proved as competent, and varietal-discriminatory metabolites. The antidiabetic potential of the different samples was explored using in-vitro amylase and glucosidase inhibition assays, which pointed out to Gwen (KG) as the most potent antidiabetic sample. This could be attributed to its enriched content of poly-unsaturated fatty acids and polyphenolics. Molecular docking was then performed to predict the most promising phytoligands in KG variety to be posed as antidiabetic drug leads. The highest in-silico α-amylase inhibition was observed with chrysoeriol-4′-*O*-pentoside-7-*O*-rutinoside, apigenin-7-glucuronide and neoeriocitrin which might serve as potential drug leads for the discovery of new antidiabetic remedies.

## Introduction

Avocado “*Persea americana* Mill.” is an evergreen subtropical tree native to southern Mexico and the Pacific Coast of Central America and is currently cultivated in many other places worldwide far from America^1^. Avocado fruit is a berry characterized by a dark green leathery skin with a very large seed. It has a high economic value in the international trade owing to its high nutrition profile and the remarkable compositional features, which are a mixture of proteins, sterols, fatty acids and coumarins^2^. Many health benefits were reported including anti-cancer, anti-oxidant and anti-microbial actions^3^.

Avocado by-products have gained a great interest as a useful agro-industrial waste. The seeds and peels are rich sources of a myriad of bioactive metabolites, including phytosterols, triterpenes, catechins, hydroxycinnamic acids, proanthocyanidins and glycosylated abscisic acid derivatives, which impart their therapeutic effects^3^. As a result, efficient, economical, and environmentally-friendly management of this agro-industrial waste constitutes a reasonable approach not only to reduce its environmental impact, but also to treat the seeds to obtain therapeutically-valuable constituents from their extract.

The chemical and biological diversities existing between the different avocado cultivars are attributed to many genetic and environmental factors enquiring scientific interest for further exploration of the avocado metabolome^2^. With the current advanced developments in analytical techniques such as LC–MS/MS and GC–MS, it has become possible to simultaneously annotate hundreds of compounds and thus identify the variance among samples^4–6^.

Diabetes mellitus is a chronic metabolic disorder characterized by hyperglycemia and the development of many vascular and neuropathic complications. It is globally ranked as the 8th chronic disease with high mortality rate. It was responsible for more than 1.4 million (2.6%) deaths in 2011 worldwide^7^. The global prevalence of type 2 diabetes increased markedly with a high level of insulin resistance, which was closely linked to the upsurge in obesity. The burden of these diseases is particularly high in middle-income countries^8^. Carbohydrate digestion has been targeted as means to manage both the postprandial elevation in blood sugar and weight gain. Indeed, *α*-amylases and *α*-glucosidases are needed for the breakdown of dietary carbohydrates into glucose. Therefore, inhibiting these enzymes is an effective strategy to lower blood glucose and control the obesity^9^. The well-known anti-diabetic drugs, clinically in use now, are *α*-glucosidase and *α*-amylase inhibitors such as acarbose, miglitol and voglibose. Nevertheless, these drugs have been reported to cause gastrointestinal side effects. In clinical treatments, acarbose, an *α*-glucosidase inhibitor that decreases postprandial hyperglycemia, may present risks associated with lower cardiovascular disease^10^. Therefore, natural inhibitors with minimal side effects can be regarded as an effective therapy for postprandial hyperglycemia^11^.

To the best of our knowledge, the discrimination between different varieties of avocado seeds using untargeted approach for metabolite profiling has not been reported yet. Thus, we aimed in the current study to investigate the varietal differences between seven studied avocado seeds collected from different geographical localities using GC/MS and LC/MS/MS analyses assisted by unsupervised pattern recognition tools. Moreover, we explored the antidiabetic action of the studied avocado varieties in-silico and in-vitro against two crucial enzymes involved in diabetes, namely *α*-amylase and *α*-glucosidase.

## Results and discussion

### HPLC–PDA/MS/MS based metabolite profiling of avocado seeds

In total, fifty-one individual metabolites were tentatively identified in the studied avocado seed hydro-ethanolic extracts via HPLC-PDA/MS/MS analysis. The UV maxima of the metabolites along with their MS/MS parent peaks, daughter fragments, elemental compositions, and distribution in the different extracts are listed in Table 1, Figure S1.

**Table 1:** Tentative identification of secondary metabolites in the studied avocado seed extracts using HPLC–PDA/MS/MS.

No	UV max	[M-H]^-^ *(m/z)*	MS^n^ product ions	El. Composition	Identification	Distribution in Avocado species							
						SA-1	LH-2	UH-3	EH-4	MH-5	RE-5	KG-7	Ref.
**Flavanols**													
1	279	288.99	145.06, 159.44, 187.11, 221.04, 245.01	C_15_H_14_O_6_	Catechin	−	−	−	−	+	−	−	^37^
2	278	289.16	159.37, 187.23, 221.06, 245.03	C_15_H_14_O_6_	Epicatechin	−	−	−	−	+	−	−	^37^
3	279	441.15	159.03, 174.75, 189.04, 203.07, 221.22, 245.08, 271.03, 289.05	C_22_H_18_O_10_	Catechin gallate	+	+	−	+	+	+	+	^37^
4	277	456.70	148.82, 159.06, 192.97, 204.05, 244.35, 269.04, 287.02, 304.74	C_22_H_18_O_11_	Epigallocatechin-3-gallate	−	+	+	−	−	+	+	^37^
5	269	305.15	158.79. 167.02, 179.02, 186.03, 203.01, 219.07, 261.03	C_15_H_14_O_7_	Gallocatechin	−	−	−	−	+	−	−	^37^
**Flavones**													
6	269, 335	563.12	353.27, 383.18, 443.16, 473.04, 503.25, 544.99	C_26_H_28_O_14_	Apigenin 6-*C*-glucopyranosyl-8-*C*-arabinopyranoside (Schaftoside)	** + **	** + **	** + **	** + **	** + **	−	−	^38^
7	271, 329	563.00	353.29, 383.14, 443.05, 473.42, 503.17, 544.82	C_26_H_28_O_14_	Isoschaftoside (Isomer of schaftoside)	** + **	** + **	** + **	** + **	** + **	−	−	^38^
8	269, 322	592.89	353.41, 383.17, 473.21, 503.25	C_27_H_30_O_15_	Apigenin-6,8-*C*-diglucoside (Vicenin 2)	** + **	−	** + **	−	** + **	−	−	^38^
9	271, 331	563.16	354.44, 443.07, 473.32	C_26_H_28_O_14_	Apigenin 8-*C*-xyloside-6-*C*-glucoside (Vicenin 3)	** + **	−	** + **	−	** + **	−	−	^38^
10	266, 325	431.14	310.89, 341.07	C_21_H_20_O_10_	Apigenin 8-*C*-glucoside (Vitexin)	−	−	** + **	−	** + **	−	−	^38^
11	266, 334	431.10	191.15, 283.74, 311.22, 341.01, 413.43	C_21_H_20_O_10_	Apigenin 6-*C*-glucoside (Isovitexin)	−	−	** + **	−	** + **	−	−	^38^
12	255, 329	592.87	294.12, 412.93	C_27_H_30_O_15_	Vitexin-4''-*O*- glucoside	−	** + **	−	−	** + **	−	−	^38^
13	269	577.43	269.04, 293.17, 402.35	C_27_H_30_O_14_	Apigenin 7-*O*-neohesperidoside (Rhoifolin)	−	−	** + **	** + **	** + **	−	** + **	^38^
14	266, 336	445.10	225.02, 269.11	C_21_H_18_O_11_	Apigenin-7-glucouronide	−	−	** + **	** + **	−	** + **	** + **	^39^
15	271, 343	447.25	327.13, 356.55	C_21_H_20_O_11_	Luteolin-8-*C*-glucoside (Orientin)	** + **	** + **	** + **	** + **	** + **	** + **	−	^38^
16	209, 254, 345	299.13	227.43, 256.14, 283.64	C_16_H_12_O_6_	Chrysoeriol	** + **	** + **	** + **	** + **	** + **	** + **	**−**	^40^
17	254, 266, 347	461.17	162.28, 298.72, 327.48, 357.21, 415.23	C_22_H_22_O_11_	Chryseriol-6-*C*-glucoside	** + **	** + **	** + **	** + **	** + **	** + **	**−**	^41^
18	255, 268, 345	623.04	161.65, 298.94, 327.23, 368.76, 399.34, 428.91, 446.95	C_28_H_32_O_16_	Chrysoeriol-*C*-hexoside- *O*-hexoside	**−**	**−**	**−**	** + **	** + **	**−**	** + **	^41^
19	255, 268, 345	607.07	161.75,282.79, 298.54, 326.87, 341.11, 429.46	C_28_H_32_O_15_	Chrysoeriol-7-*O*-neohesperidoside	**−**	**−**	** + **	**−**	** + **	**−**	**−**	^13^
20	255, 268, 345	739.49	299.23, 307.9, 327.18, 429.13, 461.0	C_33_H_40_O_19_	Chrysieriol-4′-*O*-pentoside-7′-*O*-rutinoside	** + **	** + **	** + **	** + **	** + **	**−**	** + **	^13^
21	274, 321	373.04	179.21, 314.88, 343.33	C_20_H_20_O_7_	Sinensetin	**−**	**−**	** + **	**−**	**−**	**−**	**−**	^42^
**Flavanone**													
22	261. 372	753.41	301.18, 548.22, 608.99, 651.14,705.21	C_34_H_42_O_19_	Bruteridin	** + **	** + **	** + **	** + **	** + **	**−**	**−**	^43^
23	n.d	723.25	271.12, 578.84, 621.17, 654.22, 708.34	C_33_H_40_O_18_	Melitidin	** + **	** + **	** + **	**−**	** + **	** + **	**−**	^43^
24	264, 355	595.45	286.74, 459.21	C_27_H_32_O_15_	Neoeriocitrin	**−**	**−**	** + **	** + **	**−**	** + **	** + **	^43^
25	285	271.26	253.04, 271.26	C_15_H_12_O_5_	Naringenin	** + **	**−**	**−**	** + **	**−**	**−**	**−**	^38^
26	275, 341	433.09	151.31, 270.67	C_21_H_22_O_10_	Naringenin-*O*-hexoside	**−**	**−**	** + **	** + **	** + **	** + **	**−**	^39^
27	277	579.23	177.27, 270.84, 447.18	C_27_H_32_O_14_	Naringin	**−**	** + **	** + **	**−**	**−**	**−**	** + **	^38^
28	283, 340	741.31	151.18, 270.75, 433.06, 578.83	C_33_H_42_O_19_	Narirutin-4′-*O*-glucoside	** + **	** + **	** + **	** + **	** + **	**−**	**−**	^44^
**Dihydroflavonols**													
29	285, 331	303.35	285.44	C_15_H_12_O_7_	Taxifolin (Dihydroquercetin)		** + **	** + **					^38^
30	241, 378	454.99	285.31, 303.11, 454.99	C_22_H_16_O_11_	7-*O*-galloyl taxifolin		** + **	** + **		** + **	** + **		^45^
**Biflavonoids**													
31	n.d	537.08	385.42, 443.17, 491.15	C_30_H_17_O_10_	Amentoflavone	** + **	**−**	** + **	** + **	** + **	** + **	** + **	^46^
**Naphthodianthrones**													
32	331, 547, 592	503.14	459.17, 477.31	C_30_H_15_O_8_	Hypericin	** + **	**−**	** + **	** + **	** + **	** + **	**−**	^46^
33	321, 545, 588	519.33	459.19, 477.12, 503.61	C_30_H_15_O_9_	Pseudohypericin	** + **	**−**	** + **	** + **	** + **	** + **	**−**	^46^
**Dihydrochalcones**													
34	324	435.19	166.87, 273.18, 296.64, 304.11, 389.25	C_21_H_24_O_10_	Phloridizin	−	+	+	+	+	−	−	^47^
35	252, 278	272.67	151.21, 166.83	C_15_H_14_O_5_	Phloretin	−	+	+	+	+	−	−	^47^
**Phloroglucinols**													
36	232	466.95	287.41, 329.13, 442.06	C_30_H_43_O_4_	Hyperfirin	** + **	**−**	**−**	**−**	**−**	**−**	**−**	^46^
37	236, 292	535.15	382.67, 466.18, 489.28	C_35_H_51_O_4_	Hyperforin	–	–	** + **	** + **	** + **	** + **	–	^46^
38	227, 295	548.73	397.21, 437.24, 479.16, 506.14	C_36_H_53_O_4_	Adhyperforin	** + **	**−**	** + **	** + **	** + **	** + **	**−**	^46^
**Phenolic acids and derivatives**													
39	291	311.35	149.35	C_13_H_12_O_9_	Caftaric acid	**−**	** + **	** + **	**−**	**−**	**−**	**−**	^48^
40	245, 281	331.23	331.23	C_20_H_28_O_4_	Carnosic acid	**−**	**−**	** + **	**−**	**−**	**−**	**−**	^49^
41	213, 283, 315	178.87	134.97	C_9_H_8_O_4_	Caffeic acid	** + **	**−**	**−**	**−**	** + **	**−**	** + **	^16^
42	n.d	355.43	178.88	C_15_H_16_O_10_	Caffeic acid-3-*O*-glucuronide	**−**	** + **	**−**	**−**		**−**	**−**	^49^
43	317	352.61	127.13, 135.32, 161.39,179.38,190.92	C_16_H_18_O_9_	Chlorogenic acid	**−**	**−**	** + **	**−**	** + **	**−**	**−**	^16^
44	325	352.62	135.38, 173.17, 179.11, 191.21	C_16_H_18_O_9_	Cryptochlorogenic acid	**−**	**−**	** + **	**−**	** + **	**−**	** + **	^16^
45	322	352.61	127.13, 173.42 179.38, 190.98	C_16_H_18_O_9_	Neochlorogenic acid	**−**	**−**	** + **	**−**	** + **	**−**	**−**	^16^
46	316	337.04	191.34, 172.74, 163.25	C_16_H_18_O_8_	5-*O*-*p*-Coumaroylquinic acid	**−**	**−**	**−**	**−**	** + **	**−**	**−**	^38^
47	215, 341	498.59	161.17, 163.78, 481.23	C_25_H_25_O_11_	4-*O*-caffeoyl-5-*O*-*p*-coumaroylquinic acid	**−**	**−**	**−**	**−**	** + **	**−**	**−**	^15^
48	278, 325	515.45	179.24, 191.15, 335.16, 352.71	C_25_H_24_O_12_	1,3-Dicaffeoylquinic acid	**−**	** + **	** + **	**−**	** + **	**−**	**−**	^16^
49	n.d	677.16	134.97, 161.04, 173.25	C_34_H_30_O_15_	3,4,5-tri-*O*-caffeoylquinic acid	**−**	**−**	** + **	** + **	**−**	**−**	**−**	^50^
50	222, 273	367.29	161.34, 173.25	C_17_H_20_O_9_	5-feruloylquinic acid	**−**	** + **	**−**	**−**	**−**	**−**	**−**	^50^
51	245, 299, 354	529.02	142.22, 510.78	C_26_H_27_O_12_	3-*O*-feruloyl-5-*O*-caffeoylquinic acid	** + **	** + **	**−**	**−**	**−**	**−**	**−**	^15^

(+) and (−) indicate the presence and absence of the metabolites, n.d; not detected.

Initial analyses of UV–Vis spectra showed the presence of different polyphenolic metabolites. The identified metabolites were classified according to their chemical classes into nine categories, viz., flavanols, flavones, flavanones, dihydroflavonols, biflavonoids, naphthodianthrones, dihydrochalcones, phloroglucinols, and phenolic acids.

Considering the identified flavanols; four monomeric flavan-3-ol aglycones and a flavan-3-ol conjugate were assigned in the studied extracts based on their structural feature peaks in MS^2^ annotations. According to the characterized metabolites, *C*-glycoside flavones could be considered as one of the landmarks of the avocado seed extracts, exhibiting the key fragmentation ions at *m/z* [(M-H)-150]^-^, [(M-H)-120]^-^, [(M-H)-90)]^-^, [(M-H)-60]^-^ along with or without the loss of H_2_O molecules at *m/z* [(M-H)-18].

Likewise, *C*-di-glycoside flavones, showed common daughter ions of [aglycone + 113] and [aglycone + 83]. However, in case of *O*-flavone and flavanone glycosides, the characteristic losses of 308, 162, 146 and 132 Da indicated the loss of a disaccharide structure, hexose (glucose or galactose), rhamnose, and pentose (arabinose or xylose), respectively^12,13^. Naringenin, was one of the major flavanones detected together with its glycosides; naringenin-*O*-hexoside and naringin, all sharing the aglycone fragment at *m/z* 271 with different isobaric fragments. Consistent with our previous study, naringenin was quantified in EH fruit and documented as a unique marker of this species with a promising anti-MRSA activity^14^.

Intriguingly, only two dihydro-flavonols were detected and identified as taxifolin and 7-*O*-galloyl taxifolin, with a characteristic fragment at *m/z* 285. Interestingly, only one biflavonoid was detected, and annotated as amentoflavone with (M-H) ion at *m/z* 537 and a base peak at *m/z* 385. Concerning the naphthodianthrones, hypericin, and pseudohypericin were detected, both sharing a characteristic base peak at *m/z* 459. Along with two dihydrochalcones, i.e., phloridizin and phloretin were identified according to their fragmentation patterns and molecular ion at *m/z* 435 and 272, respectively. In addition, three phloroglucinols; hyperfirin, hyperforin, and adhyperforin, were assigned according to their molecular ion peaks at *m/z* 467, 535 and 549, respectively. To the best of our knowledge, this is the first report about the identification of amentoflavone, phloroglucinols, and naphthodianthrones in avocado seeds.

Structurally, quinic acid was the major phenolic acid that was tentatively identified in the investigated samples. Six derivatives of quinic acid were annotated based on the presence of the diagnostic ion at *m/z* 191^15^. Unambiguous identification of caffeic acid and its derivatives was established based on their base peak at *m/z* 179, which undergoes decarboxylation to produce *m/z* 135 [M-H-H_2_O-CO]^-^. Despite the structural similarity of the three isomers of chlorogenic acids, they showed a characteristic base peak at *m/z* 353 with quite different abundance of the fragmentation ions. As previously reported, this diagnostic fragment was used to discriminate between them^16^. In line with the findings of Di Stefano et al. (2016), who discovered significant variations in the metabolic profiles of Italian avocado cultivars. Chlorogenic acid, 4-hydroxybenzoic acid, and protocatechuic acid were detected in the ripe fruits. On the contrary, only epicatechin tended to decrease along with fruit ripening^17^.

### GC/MS metabolite profiling of avocado seeds

The analysis of un-saponified and saponified compounds detected in the avocado samples by GC/MS led to the identification of 68 un-saponified and 26 saponified compounds. Table 2 illustrates that saturated aliphatic hydrocarbons were the major constituents among all the samples represented by tetradecane, hexadecane, pentadecane, and trimethyl dodecane. Egyptian reed (ER) and Morocco (MH) and Lebanese Hass (LH) were the most abundant seeds with hydrocarbons. Whereas, EH (Egyptian Hass) and KG (Gwen avocado from Kenya) showed minor content of aliphatic hydrocarbons.

**Table 2:** Relative percentage of the un-saponified compounds in the studied avocado seed extracts using GC/MS.

No	Compounds	Rt (min)	SA	LH	UH	EH	MH	RE	KG
**Saturated aliphatic hydrocarbons**									
1	Dodecane	16.13	1.41	−	−	−	−	−	−
2	Undecane, 2,6-dimethyl	16.36	0.86	−	−	−	−	0.6	−
3	Tridecane	17.53	0.77	−	−	−	−	−	−
4	Tridecane, 7-methyl-	18.0	3.54	−	−	−	−	−	−
5	Dodecane, 4,6-dimethyl-	18.81	−	−	−	−	1.26	2.69	0.96
66	Tridecane	18.88	7.33	1.32	−	−	−	8.79	1.69
7	Tetradecane	19.26	1.2	−	−	−	−	1.03	−
8	Tetradecane, 2,6,10-trimethyl	19.76	−	0.95	−		2.7	−	−
9	Eicosane, 10-methyl-	20.58	2.27	1.14	−	−	−	−	−
10	Dodecane, 2,6,10-trimethyl-	20.82	8.62	7.11	2.95	1.43	10.11	7.46	2.57
11	Tetradecane	21.57	**15.49**	**13.92**	6.62	2.17	**17.25**	**20.78**	5.07
12	Octadecane, 1-chloro-	22.8	0.73	1.19	0.47	−	0.79	−	0.53
13	Hexadecane	23.05	5.58	**11.08**	5.39	−	**10.4**	5.58	−
14	Heptadecane, 2,6,10,14-tetramethyl-	23.36	−	−	−	2.68	−	−	1.45
15	Tetradecane, 2,6,10-trimethyl-	23.41	2.35	0.9	1.55	−	1.04	−	−
16	Tetradecane, 3-methyl-	23.88	−	3.63	−	−	−	−	−
17	Tetradecane, 2-methyl-	23.99	−	1.01	−	−	1.83	1.34	−
18	Heptadecane	24.03	−	1.99	−	−	−	−	−
19	Pentadecane	24.18	5.67	**11.63**	6.01	6.01	8.0	7.76	2.28
20	Dotriacontane	26.31	−	−	0.58	−	1.02	−	−
21	Pentacosane	44.56	−	0.67	−	−	−	−	−
22	Tetratetracontane	47.43	−	0.64	0.52	1.05	−	−	0.4
23	Octadecane, 3-ethyl-5-(2-ethylbutyl)-	50.5	−	0.67	−	0.71	0.89	−	−
Total saturated aliphatic hydrocarbons			55.82	57.18	24.09	13.34	54.4	56.03	14.95
**Unsaturated aliphatic hydrocarbons**									
24	Cyclohexene, 3-(1-hexenyl)	33.71	−	−	−	5.57	−	−	−
25	Cyclohexene, 3-(3-methyl-1-butenyl)	37.31	−	−	**11.73**	9.83	−	−	**10.81**
26	1-Nonadecene	35.73	−	−	−	2.4	−	−	−
27	5-Eicosyne	36.1	−	1.92	3.83	−	−	−	−
28	10-Heneicosene	42.97	−	−	−	1.03	−	−	−
Total Unsaturated aliphatic hydrocarbons			−	1.92	15.56	18.83	−	−	10.81
**Monoterpene hydrocarbons**									
29	l-Limonene	12.66	−	−	−	−	1.15	1.2	0.71
30	à-Humulene	23.8	−	−	−	−	−	−	0.86
31	Total monoterpene hydrocarbons		−	−	−	−	1.15	1.2	1.57
32	Sesquiterpene hydrocarbons								
33	à-Cubebene	20.39	3.9	−	−	−	−	−	3.66
34	α -Copaene	21.62	−	−	−	−	−	−	1.53
35	Trans-Caryophyllene	22.52	1.09	−	−	−	−	−	−
36	Trans-à-Bergamotene	22.66	3.11	−	−	−	−	−	−
37	á-elemene	32.26	2.52	−	−	−	−	1.73	**8.51**
Total sesquiterpene hydrocarbons			10.62	−	−	−	−	1.73	13.7
**Oxygenated sesquiterpenes**									
38	Caryophyllene oxide	26.82	1.23	−	1.55	2.73	−	−	1.22
39	Isoaromadendrene epoxide	30.16	−	−	−	1.27	−	−	−
Total oxygenated sesquiterpenes			1.23	−	1.55	4.0	−	−	1.22
**Alcohols**									
40	2-Methyl-cetyl alcohol	22.79	−	0.86	−	−	0.82	−	−
41	Cetyl alcohol	31.68	−	−	−	1.92	−	−	−
42	Trans-Geranylgeraniol	32.88	−	−	−	−	1.83	−	−
43	epi-á-Santalol	33.24	6.41	−	−	−	−	−	7.27
44	α-Santalol	34.02	−	−	−	−	−	−	1.08
45	Phytol	38.1	0.49	−	0.49	2.61	−	−	−
46	Ethanol, 2-(9,12-octadecadienyloxy)	35.75	−	−	0.58	3.94	0.99	0.93	1.88
Total alcohols			6.9	0.86	1.07	6.55	3.64	0.93	11.45
**Ethers**									
47	7-Dodecynyl tetrahydro-2H-pyran-2-yl ether	40.62	1.06	−	−	−	−	−	−
Total ethers			1.06	−	−	−	−	−	−
**Epoxides**									
48	Humulene oxide or Humulene epoxide I	27.74	−	−	−	−	−	−	0.53
Total epoxides			−	−	−	−	−	−	0.53
**Aldehydes/ketones**									
49	Pregan-20-one,2-hydroxy-5,6-epoxy-15-methyl	29.29	−	−	−	0.6	−	−	−
50	9,17-Octadecadienal	31.98	−	−	**8.71**	−	−	−	7.89
51	Bicyclo[3.2.2]nona-2,6-dien-5-ol-4-one	33.87	−	−	−	−	4.2	−	−
52	Iso-jasmone	36.81	−	−	2.29	−	−	−	−
53	Koiganal II	35.71	1.37	−	−	−	−	−	−
Total aldehydes/ketones			1.37	−	11.0	0.6	4.2	−	7.89
**Esters**									
54	Ethyl iso-allocholate	27.3	−	1.36	−	0.52	1.6	−	−
55	Omega-3-arachidonic acid methyl ester	27.46	−	−	0.85	−	−	−	−
56	NerolidoL-epoxyacetate	30.22	−	−	−	−	−	0.71	−
57	3,13-Octadecadien-1-ol acetate	31.44	7.72	−	−	−	−	−	−
58	3,7,11,Trimethyl-8,10-dodecedienylacetate	31.97	−	−	−	**13.61**	−	−	−
59	Methyl-8,11,14,17-eicosatetraenoate	32.69	−	−	−	**10.05**	−	−	−
60	3,15-Octadecadien-1-ol acetate	32.02	−	1.64	−	−	−	−	−
61	Methyl 5,9,12-octadecatrienoate	44	−	−	−	−	−	1.37	−
62	Methyl -5,11,14,17-eicosatetraenoate	40.48	1.85	3.12	10.86	6.22	1.55	6.58	**16.2**
63	Ethyl linoleate	43.56	−	−	−	−	−	−	2.18
64	Cyclo propaneoctanoic acid,2-[[2-[(2-ethylcyclopropyl)methyl] cyclopropyl]methyl]-, methyl ester	43.81	−	2.1	−	−	−	−	−
Total esters			9.57	6.86	11.71	30.4	3.15	8.66	18.38
**Acyclic carotene**									
65	Lycopersen	49.44	−	−	−	0.68	−	−	−
Total acyclic carotene			−	−	−	0.68	−	−	−
**Sterols**									
66	Campesterol	57.98	−	−	−	0.64	−	−	−
67	Sitosterol	59.51	1.57	2.2	2.1	6.89	2.02	0.61	1.14
Total sterols			1.57	2.2	2.1	7.53	2.02	0.61	1.14
**Vitamins**									
68	1,25-dihydroxy vitamin D2	42.65	−	−	−	0.55	−	−	−
Total vitamins			−	−	−	0.55	−	−	−

The main compounds for each species are underlined and bolded.

The separation was performed on TG-5MS column (30 m × 0.25 mm i.d., 0.25 μm film thickness).

Aliphatic esters constituted the second major class identified among un-saponified compounds in seed samples. Methyl -5,11,14,17-eicosatetraenoate was the main ester in KG accounting for 16.2% of the total identified compounds. Aliphatic alcohols, aldehydes, and ketones were also identified in many samples. Santalol was the representative sesquiterpenoid alcohol detected in a relatively high concentration in both SA (Pinkerton avocado of South Africa) and KG only. Importantly, *α*-santalol, the major constituent of sandalwood oil, showed efficient antioxidant and anti-hyperglycemic activity in alloxan-induced diabetic animal models^18^.

Regarding the saponified compounds, fatty acid methyl esters (FAME) were the major compounds detected among all samples especially methyl linoleate and methyl oleate. Table 3 illustrates that methyl linoleate (18:2 omega 6) was the most abundant ester among the samples. Their concentrations were in the following order: KG > RE > EH > SA > UH > MH > LH. It constituted 63.24% of the total identified compounds in KG. Therefore, it could be considered as a valuable source of healthy omega-6 fatty acid. As primary precursors of lipid mediators, both omega-6 fatty acid and omega-3 arachidonic acid methyl esters are considered important structural elements of cell membranes^19^.

**Table 3:** Relative percentage of the saponified compounds in the studied avocado seed extracts using GC/MS.

No	Compounds	Rt (min)	SA	LH	UH	EH	MH	RE	KG
**Esters**									
1	Oxiraneun decanoic acid, 3-pentyl-methyl ester	26.89	0.16	−	−	−	0.16	−	−
2	Cyclooctasiloxane, hexadecamethyl	27.45	−	−	−	0.09	−	−	−
3	Tridecanoic acid, methyl ester	28.48	0.37	0.17	0.06	0	0.58	0.21	0.09
4	Methyl tetradecanoate	31.18	0.32	0.7	0.15	0.53	0.85	0.36	0.35
5	Pentadecanoic acid, methyl ester	33.92	0.12	0.3	0.09	0.28	0.45	0.15	0.09
6	13,16-Octadecadiynoic acid, methyl	34.06	0.09	−	−	−	−	0.1	−
7	Methyl arachidonate	34.51	−	−	−	−	−	−	0.1
8	Methyl palmitoleate	35.98	0.72	2.17	1.2	2.22	2.79	0.35	0.76
9	Methyl-7)-Hexadecenoate	36.23	23.45	−	−	−	−	2.14	−
10	Methyl palmitate	36.52	0.62	**44.79**	22	23.65	25.89	21.59	17.18
11	Methyl isostearate	39.14	**33.68**	0.26	0.17	0.55	0.23	−	0.08
12	9,12-Octadecadienoyl chloride ,	40.08	−	−	−	−	−	0.56	0.17
13	9,12-Octadecadienoic acid), methyl ester(methyl linoleate)	40.82	30.99	13.22	28.72	**35.91**	17.96	**44.38**	**63.24**
14	Methyl oleate	41.11	1.48	24.33	**37.97**	26.98	**37.09**	25.16	12.81
15	13-Tetradecynoic acid, methyl ester	41.85	−	−	−	0.61	−	0.3	0.33
16	Eicosanoic acid, methyl ester	46.43	0.12	0.37	0.29	0.91	0.91	−	−
17	13-Docosenoic acid, methyl ester	50.71	0.59	0.59	0.58	0	−	−	0.17
18	Docosanoic acid, methyl ester	50.76	−	−	−	−	0.15	0.38	0
19	Eicosanoic acid, methyl ester	52.77	−	−	0.83	0.35	1.93	0.07	0.13
20	Tetracosanoic acid, methyl ester	54.92	0.84	2.92	1.06	−	2.92	0.55	0.53
21	Cyclo pentanetridecanoic acid, methyl ester	58.76	0.42	0.05	0.31	−	0.37	0.12	0.12
22	Octacosanoic acid, methyl ester	62.4	0.17	0.25	0.13	0.31	−	−	−
Total esters			94.14	89.87	93.56	92.39	92.28	96.42	96.15
**Monoterpenes hydrocarbons**									
23	Terpinene	10.5	−	−	−	0.17	−	−	−
Total monoterpenes hydrocarbons			−	−	−	0.17	−	−	−
**Sesquiterpene hydrocarbons**									
24	Trans-calamenene	25.58	−	−	−	−	−	0	0.09
Total sesquiterpene hydrocarbons			−	−	−	−	−	0	0.09
**Miscellaneous compounds**									
25	3-Butoxy-1,1,1,7,7,7-hexamethyl-3,5,5 tris(trimethylsiloxy)tetrasiloxane	21.87	−	0	0	0.1	−	−	−
26	1-Hydroxy-6-(3-isopropenyl-cycloprop -1-enyl)-6-methyl-heptan-2-one	26.41	−	−	−	−	−	0.08	−

The separation was established on TG-5MS column (30 m × 0.25 mm i.d., 0.25 μm film thickness).

The main compounds for each species are underlined and bolded.

Consistent with our results, a recent report by Soledad et al., 2021 demonstrated that methyl esters of linoleic, and linolenic acids were the predominant phytochemicals in Mexican avocado seeds. *β*-sitosterol content was significantly varied (0.61–6.89%) among the seed samples^19^. Sitosterol, stearic and linoleic acids were the most predominant compounds in the ripe fruits of Spanish avocado cultivars^20^. In contrast, Salazar-López et al., 2020 reported that stigmast-5-en-3-ol was the main phytosterol of Hass avocado^21^.

More recently, methyl lineoleate, *β*-sitosterol and campesterol were reported to ameliorate the glucose level in sucrose induced diabetes in rats by regulation of glucose transporter proteins and insulin receptor due to their lipophilic nature^22^. Hydrocarbons and esters were important volatile components of avocado that contributed to the quality and flavor of the fruits^21^. Monoterpene and sesquiterpene hydrocarbons were identified as minor constituents among all samples. *α*-elemene, was the most important sesquiterpene hydrocarbons identified in KG with the highest threshold (8.51%). To the best of our knowledge, this study represents the first comprehensive volatile profiling of the seven species of avocado seeds belonging to different geographic origins.

### Avocado varietal discrimination via PCA and HCA analysis of GC/MS data matrix

Owing to the complexity of the acquired data, multivariate data analysis, viz. HCA and PCA were performed to define both similarities and differences amongst the studied avocado seed samples. The metabolic variability of the different avocado seed varieties was characterized using untargeted GC/MS based metabolomics assisted by multivariate data analyses. Data matrices obtained from the combined un-saponified and saponified seeds metabolites were employed.

**Figure 1:**
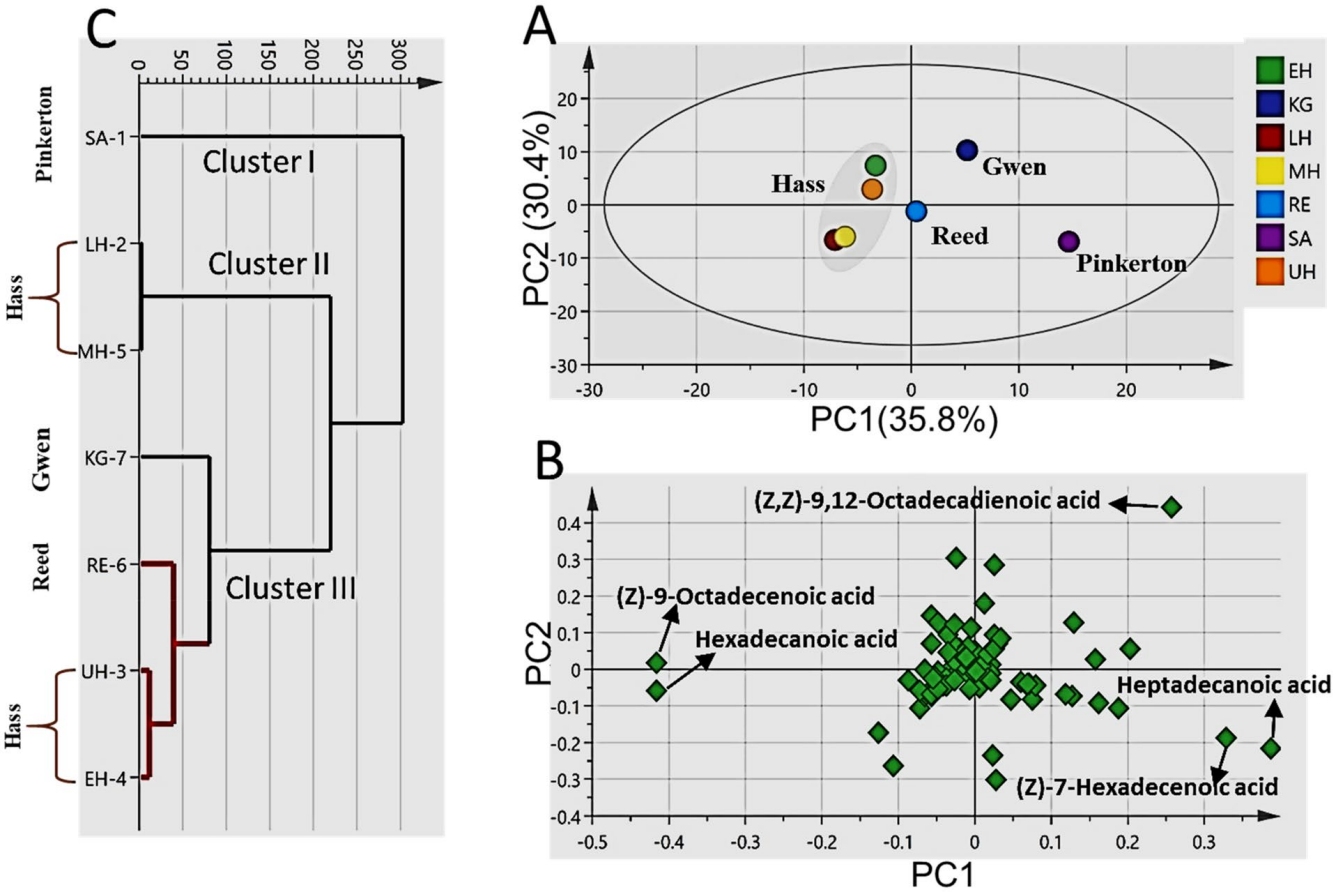
Principal component analysis and hierarchical clustering of the studied avocado seed extracts based on the combined GC/MS datasets derived from saponified and unsaponified metabolites. (**A**) Score plot of PC1 vs. PC2 scores. (**B**) Loading plot for PC1 & PC2 contributing metabolites and their assignments. (**C**)- HCA dendrogram.

PCA of the combined GC/MS datasets produced a model (Fig. 1) prescribed by PC1/PC2 accounting for 66% of the variance in compositional makeup among the different seed varieties. Score plot (Fig. 1A) revealed the general structure of the dataset and the samples grouping where Pinkerton variety represented by “SA” sample obtained from South Africa was located distant from the other samples. The most influential metabolites responsible for this separation were revealed from the PCA loading plot (Fig. 1B) as being (Z)-7-hexadecenoic acid and heptadecanoic acid, as implied by their high factor loadings over the positive side of PC1, which were more abundant in “SA” sample. Gwen variety “KG sample obtained from Kenya”, located in the upper right side of the PCA score, was shown to be the most enriched in 9,12-octadecadienoic acid (linoleic acid). However, the four Hass varieties “EH, LH, UH & MH” obtained from Egypt, Lebanon, Morocco, and USA clustered close to each other and found more abundant in hexadecanoic (palmitic acid) and 9-octadecenoic acids.

HCA, another unsupervised pattern recognition method with different graphical presentation revealed a comparable varietal classification to PCA. HCA-derived dendrogram (Fig. 1C) depicted three clusters (I–III), in which Pinkerton variety “SA” was clustered in a separate cluster exhibiting a long distance compared with the other samples on the cluster scale. Cluster II included 2 Hass varieties *i.e.,* “LH and MH” avocado samples obtained from Lebanon and Morocco while cluster III included Gwen variety “KG sample from Kenya” and the other sub-tree contained Reed variety “RE sample from Egypt” and the other two Hass samples “UH and EH collected from USA and Egypt” indicating their comparable fatty acid profile.

From the PCA & HCA analyses, it can be concluded that the variabilities in fatty acid accumulation in the studied seed samples obtained from a wide regional divergence were efficient in the discrimination between the different seed varieties.

### Antidiabetic activity of avocado seeds

Diabetes is a chronic metabolic syndrome and is considered a life- threatening disease characterized by the inability of the pancreas to secrete insulin or insulin resistance. Uncontrolled hyperglycemia is directly associated with a high risk of diabetic related complications such ischemic heart disease, stroke, and diabetic retinopathy. According to a recent estimate by the World Health Organization (WHO), more than 422 million patients suffered from diabetes worldwide in 2014 and the prevalence of the disease threatening will increase dramatically by 2040 to exceed 642 million^23^.

*α*-glycosidase and *α*-amylase are the key enzymes which play an essential role in the dietary digestion of carbohydrates. Enzyme inhibitors were believed to be the most effective strategy for the management of diabetes and reducing its complications. Unfortunately, acarbose, the first approved enzyme inhibitor suffered from unexpected side-effects such as stomach-ache, flatulence, and diarrhea^24^. Recently, the trends toward natural phytoconstituents as enzyme inhibitors have gained a special interest in many scientific studies over the synthetic hypoglycemic agents^23,24^. As shown in Table S2, Gwen avocado (KG) showed the highest inhibitory activity against both *α*-glycosidase and *α*-amylase with IC_50_ of 55.07 ± 2.48 and 95.3 ± 2.02 µg/ml, respectively, compared to acarbose. On the other hand, Egyptian reed (RE) showed the lowest glucosidase inhibitory activity, with almost no significant activity against amylase.

Our results are shedding the light on understanding the vital role of polyunsaturated fatty acids such as linoleic acid, and its methyl ester as antidiabetic compounds identified in a high concentration in KG (*ca*. 63.24%) of the saponified compounds. In agreement with a recent study of Smorowskaet al., 2021 that highlighted the potential activity of oleic and linoleic fatty acids rich fraction of blue corn extract as *α*-amylase inhibitor^25^.

In the same line, *β*-sitosterol identified in *Syzygium cumini* L. leaves was responsible for the antidiabetic activity^26^. α-Humulene, a natural monocyclic sesquiterpene, and 9,12-octadecadienoic acid methyl ester as well as linoleic acid esters, identified in KG extract, were previously reported as natural antioxidants with antidiabetic and neuroprotective activities in the essential oil of *Elaeagnus umbellata* Thunb. fruits^27^.

In addition, epigallocatechin-3-gallate (EGCG) was identified by HPLC-MS/MS analysis as a unique component in KG extract which may contribute to its potential anti-diabetic activity. In a randomized placebo-controlled clinical study, catechins of green tea especially EGCG demonstrated a non-competitive *α*-glucosidase inhibitory activity higher than acarbose^28^. Recently, many polyphenolic compounds have been detected in cereals as quercetin, kaempferol, luteolin, naringenin and apigenin glycosides, which were reported as potent inhibitors of both amylase and glucosidase enzymes^29^.

Ultimately, the synergistic activity of both major and minor bioactive metabolites in KG was responsible for its potential activity as an amylase and glucosidase inhibitors.

### Virtual screening of avocado phytoligands as potential *α*-amylase inhibitors

**Figure 2:**
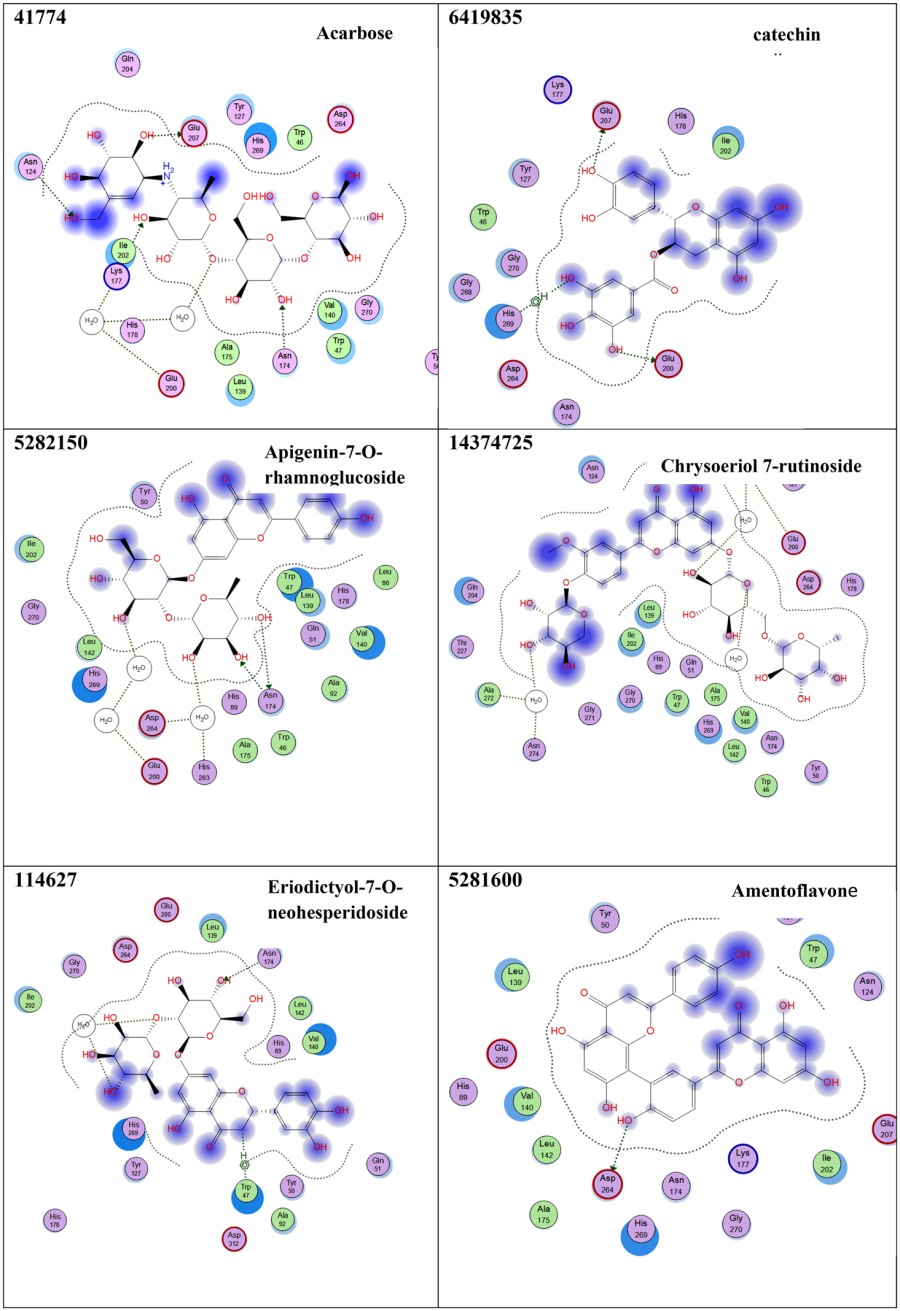

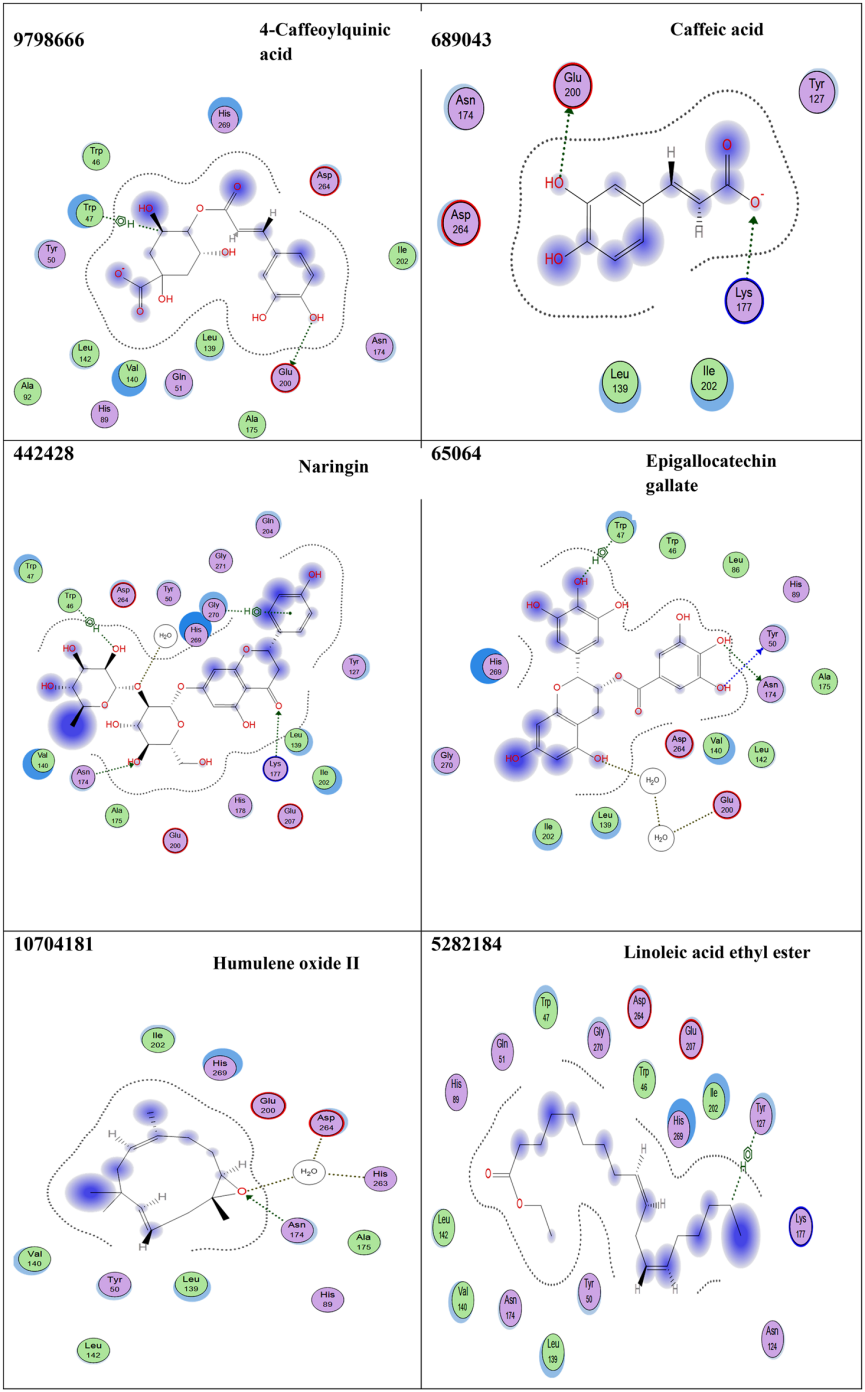

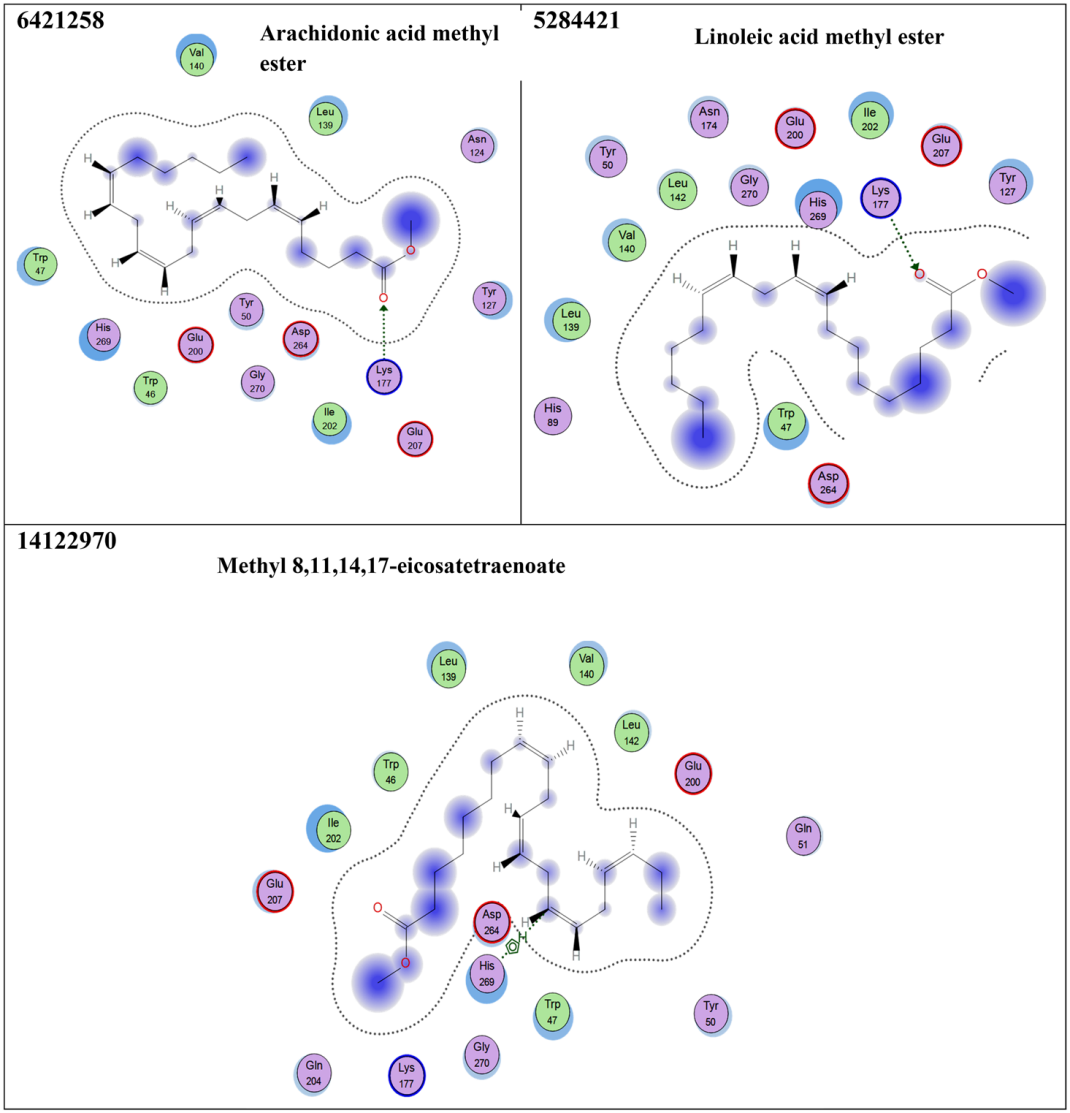
Interaction diagrams of the docked phytoligands i.e., 15 compounds with the active sites of α-amylase (PDB: 1Kxh). Green arrow represents side chain acceptor/donor; blue arrow represents backbone acceptor/donor; blue shadow represents ligand exposure. The studied phytoligands are depicted in Table 4.

Sixteen compounds were docked into the active site of *α*-amylase, a crucial enzyme involved in diabetes (Fig. 2 and Table 4). The docking simulations results showed that apigenin-7-*O*-rhamnoglucoside exhibited the highest α-amylase inhibitory activity, possessing a binding score of − 20.58 kcal/mol which was higher than that of acarbose (− 19.40 kcal/mol), followed by naringenin which exhibited a binding score of – 20.06 Kcal/mol. Other compounds displayed a relatively low binding energy compared to the reference drug, acarbose. These include catechin gallate, chrysoeriol 7-rutinoside, eriodictyol-7-*O*-neohesperidoside, and epigallocatechin gallate. They also shared similar interactions like those shown from acarbose such as Glu 200 (Hydrogen bonding), Glu 207 (Hydrogen bonding), and Asn 174 (Hydrogen bonding). A moderate inhibitory activity on amylase enzyme was observed by amentoflavone**,** caffeic acid, and 4-caffeoylquinic acid, (Fig. 2 and Table 4). Accordingly, 15 phytoligands Fig. 2 showed strong interactions with the active site residues of the *α*-amylase enzyme. As a result, they might be considered as the potential drug candidates for the discovery of novel *α*-amylase inhibitors after they are screened for their safety profile and efficacy via in-vivo and clinical studies.

**Table 4:** Docking simulations results of the studied phytoligands identified by GC/MS and HPLC-MS/MS analysis, respectively using acarbose as a co-ligand.

CID	phytoligands	Free energy of binding (∆G) (Kcal/mol)	Interactions at the binding interface
41,774	Acarbose	− 19.40	Glu 200 (Hydrogen bonding through solvent) Glu 207 (Hydrogen bonding) Asn 124 (Hydrogen bonding) Asn 174 (Hydrogen bonding) Lys 177 (Hydrogen bonding)
6,419,835	Catechin gallate	− 19.12	Glu 200 (Hydrogen bonding) Glu 207 (Hydrogen bonding) His 269 (Hydrophobic interaction)
5,282,150	Apigenin-7-*O*-rhamnoglucoside	− 20.58	Glu 200 (Hydrogen bonding through solvent) Asn 174 (Hydrogen bonding) Asp 264 (Hydrogen bonding through solvent) His 263 (Hydrogen bonding through solvent)
14,374,725	Chrysoeriol 7-rutinoside	− 18.96	Glu 200 (Hydrogen bonding through solvent) Asn 274 (Hydrogen bonding through solvent) Ala 272 (Hydrogen bonding through solvent)
114,627	Eriodictyol-7-*O*-neohesperidoside	− 19.54	Asn 174 (Hydrogen bonding) Trp 47 (Hydrophobic interaction)
5,281,600	Amentoflavone	− 14.19	Asp 264 (Hydrogen bonding)
9,798,666	4-Caffeoylquinic acid	− 13.79	Glu 200 (Hydrogen bonding) Trp 47 (Hydrophobic interaction)
689,043	Caffeic acid	− 14.64	Glu 200 (Hydrogen bonding) Lys 177 (Hydrogen bonding)
442,428	Naringin	− 20.06	Lys 177 (Hydrogen bonding) Asn 174 (Hydrogen bonding) Gly (Hydrophobic interaction) Trp 46 (Hydrophobic interaction)
65,064	Epigallocatechin gallate	− 18.21	Asn 174 (Hydrogen bonding) Trp 47 (Hydrophobic interaction) Glu 200 (Hydrogen bonding through solvent) Tyr 50 (Hydrogen bonding)
5,281,520	Alpha-Humulene	Failed	
10,704,181	Humulene oxide II	− 10.81	Asn 174 (Hydrogen bonding) Asp 264 (Hydrogen bonding through solvent) His 263 (Hydrogen bonding through solvent)
5,282,184	Linoleic acid ethyl ester	− 9.07	Tyr 127 (Hydrophobic interaction)
6,421,258	Arachidonic acid methyl ester	− 8.80	Lys 177 (Hydrogen bonding)
5,284,421	Linoleic acid methyl ester	− 9.58	Lys 177 (Hydrogen bonding)
14,122,970	Methyl 8,11,14,17-eicosatetraenoate	− 8.12	His 269 (Hydrophobic interaction)

*The best ligand-receptor complex binding free energy at RMSD < 2 .

## Conclusion

Agro-industrial by-products represent a global challenge due to their impact on economic, social, and environmental sectors. The green recycling of these by-products poses a crucial strategy to fight diseases. In our study, compositional differences existing among the investigated avocado varieties were assessed using GC/MS and HPLC–MS/MS analysis assisted by unsupervised pattern recognition tools. Gwen seeds exhibited the highest antidiabetic potential against two diabetic marker enzymes*,* i.e., α-amylase and α-glucosidase, compared to acarbose drug. This activity could be attributed to the presence of high levels of polyunsaturated fatty acids as well as several polyphenolic compounds as revealed by the molecular docking study.

Our findings revealed that Gwen's variety of avocado represents a promising, low cost, and readily available natural source of bioactives to be used as an antidiabetic remedy. Further studies are still required for the successful commercial use of Gwen avocado by-products as a sustainable source of antidiabetic metabolites. Moreover, comprehensive studies are still required to investigate the effect of fruit ripening on the metabolic profile of the seeds, especially Gwen cultivar.

## Material and methods

### Plant material

Seven well characterized samples of avocado fruits were purchased in June 2016 from different geographical localities across the world. Three avocado varieties, i.e., Reed, Pinkerton and Gwen were obtained from Egypt, South Africa, and Kenya, respectively. In addition to, Hass cultivar was obtained from Egypt, Lebanon, USA and Morocco. They were selected at the full ripening stage. All fruits were collected and identified with permission from the Agriculture Research Center, Giza, Egypt at "9 Cairo University Road, Giza District, Giza Governorate". The fruit collection process has been established according to the national guidelines. The plant material was kindly validated by Dr. Essam Abel Karim (Tropical Fruit Research Institute, Giza, Egypt).

The voucher specimens were deposited at Faculty of Pharmacy Herbarium, Cairo University, under the identification numbers listed in Table S1. The seeds were separated from their fruit and weighted using a sensitive balance. The variation in weights was listed in Table S1. They were dried at 50 °C and grounded to fine powder. The powdered seeds (5 gm) were defatted using hexane (3 × 150 ml), which were then extracted with methanol (3 × 100 ml). Hexane extracts of all samples were subjected to esterification for the preparation of fatty acid methyl ester (FAME). For HPLC-MS/MS analysis, powdered seeds (10 mg) were extensively extracted with methanol. The solvents were evaporated under reduced pressure using Buchi®R-300, USA.

### Chemicals and reagents

All chemicals such as methanol, hydrocarbons (HC) and DMSO (LC-MS/MS grade), fatty acid methyl esters (FAME) standards and acarbose (≥ 95%) were purchased from Sigma–Aldrich (St. Louis, MO, USA). Similarly, *Saccharomyces cerevisiae α*-glucosidase (EC 3.2.1.20) of 0.05 U/ml (type I, ≥ 10 units/mg protein) and *α*-amylase (EC 3.2.1.1) (type VI-B, ≥ 10 units/mg solid) used for biological assays were obtained from Sigma–Aldrich (St. Louis, MO, USA). Other chemicals, viz., sodium hydroxide, sodium chloride, sodium acetate, sodium potassium tartrate, di-nitro salicylic acid solution, sucrose, Tris buffer pH 8.0, and boron trifluoride (BF_3_) were of analytical grade and were obtained from El-Gomhouria Co. for trading chemicals and medical supplies (Cairo-Egypt).

### High-performance liquid chromatography-tandem mass spectrometer (HPLC-MS/MS)

The LC system was Thermo Finnigan (Thermo Electron Corporation, Waltham, MA, USA) equipped with a reversed-phase column (Zorbax Eclipse XDB-C18, 4.6 × 150 mm, 3.5 µm, Agilent, Santa Clara, CA, USA) as described before^30^. Briefly, LC instrument was connected to the mass spectrometer (LCQ-Duo ion trap) with an electrospray ionization source. Water and acetonitrile (ACN) with 0.1% formic acid in both solvents were used to create a gradient flow of the mobile phase. A gradual increment of ACN, from 5 to 30% for 60 min and increased to 90% in the last 30 min, was performed at a flow rate of 1 ml/min with a split ratio of 1:1. The full scan acquisition method was established, and the spectra scan range was acquired at *m/z* 50–2000 in the negative mode. The browser X-calibur software (X-calibur ™ 2.0.7, Thermo Fisher Scientific, Waltham, Ma, USA) was used to evaluate the data.

### Gas chromatography/mass spectrometry (GC/MS) for preparation of fatty acid methyl ester (FAMEs)

The GC/MS system was equipped with a TG-5MS column (30 m × 0.25 mm i.d., 0.25 μm film thickness). Helium was used as carrier gas at for the analysis of the methyl esters of fatty acids. The analysis was established using a TRACE GC Ultra Gas Chromatograph (THERMO Scientific Corp., USA), was coupled with a thermo mass spectrometer detector (ISQ Single Quadrupole Mass Spectrometer). Helium was used as the inert carrier gas at a flow rate of 1.0 ml/ min and a split ration of 1:10. The temperature was programmed as follows: 80 °C for 1 min; raised at 4.0 °C/min to 300 °C and held for 5 min. A volume of 0.2 μl of the diluted samples (1:10 hexane, v/v) was injected. The injector and the detector were both kept at 240 °C. Mass spectra were obtained by electron ionization (EI) at 70 eV, using a spectral range of m/z 40–450. Samples containing fatty acids were esterified to more volatile methyl esters by methanol:BF_3_ as previously described^31^.

### GC/MS of the un-saponified fraction

The same spectrophotometric parameters were established as described in previous section except in the following: the temperature was programmed at 55 °C for 1 min; raised at 5.0 °C/min to 300 °C and held for 15 min. Both the injector and detector temperatures were held at 280 °C. Silylated compounds were identified by GC/MS volatile analysis as previously described and their contents were calculated based on peak areas relative to summed peak areas of identified metabolites.

### Multivariate statistical analysis

The relative peak areas of the phytochemicals identified by GC/MS analysis were subjected to exploratory analysis using SIMCA-P version 13.0 software package (Umetrics, Umeå, Sweden). An exploratory analysis of the data was carried out using principal component analysis (PCA) and hierarchical clustering analysis (HCA), which were used to explore the metabolic profiles' heterogeneity/similarity between the different avocado samples based on their varietal region. All variables were mean-centered and scaled to Pareto variance^32^.

### In-vitro *α*-amylase inhibition assay

The in-vitro *α*-amylase inhibition assay was quantitatively assayed by a colorimetric method according to the previously described method^33^.

### In-vitro *α*-glucosidase inhibition assay

The in-vitro *α*- glucosidase inhibitory activity was determined using a spectrometric based assay according to the method established by Pandithurai et al.^33^.

### Molecular docking of avocado phytoligands

The docking simulations were performed, using Molecular Operating Environment (MOE) software package version 2013.08; (available from Chemical Computing Group Inc., Montreal, QC, Canada) on the phytochemicals characteristic to the avocado sample (KG) showing the highest in-vitro* α*-amylase activity as identified by GC/MS and HPLC-MS/MS analysis. Three-dimensional (3D) structures of all the studied phytoligands were retrieved from the PubChem database (http://pubchem.ncbi.nlm.nih.gov), which were then docked into the rigid *α*-amylase binding pocket. The 3D crystal structure of *α*-amylase was downloaded from Protein Data Bank (PDB code: 1KXH) bound to acarbose. The structure of chain A was processed using the Structure Preparation application in MOE^34^. The docking protocol was followed as previously applied in^35,36^.

## Supplementary Information

Supplementary Information.

## Data Availability

All data generated or analysed during this study are included in this published article (and its Supplementary Information files).
