# Supplementary material for: Metabolomics-based profiling of 4 avocado varieties using HPLC–MS/MS and GC/MS and evaluation of their antidiabetic activity
Source: Sci Rep. 2022 Mar 23;12:4966. doi: 10.1038/s41598-022-08479-4 (PMC8943142; doi:10.1038/s41598-022-08479-4)

Supplementary material

**Metabolomics-based profiling of 4 Avocado varieties using HPLC-MS/MS and GC/MS and evaluation of their antidiabetic activity**

**Inas Y. Younis^a,1*^, Amira R. Khattab^b,1^,** **Nabil M. Selim^a^, Mansour Sobeh^c*^, Seham S. Elhawary^a^, Mahitab H. El Bishbishy^d^**

**^a^** Pharmacognosy Department, Faculty of Pharmacy, Cairo University, Cairo 11562, Egypt

[inas.younis@pharma.cu.edu.eg](mailto:inas.younis@pharma.cu.edu.eg), [nabil.selim@pharma.cu.edu.eg, seham.elhawary](mailto:nabil.selim@pharma.cu.edu.eg,%20%20seham.elhawary) @pharma.cu.edu.eg

^b^ Pharmacognosy Department, College of Pharmacy, Arab Academy for Science, Technology and Maritime Transport, Alexandria 1029, Egypt; [Dr_amira_khattab@aast.edu](mailto:Dr_amira_khattab@aast.edu).

^c^ AgroBioSciences, Mohammed VI Polytechnic University, 43150 Ben-Guerir, Morocco, [Mansour.sobeh@um6p.ma](mailto:Mansour.sobeh@um6p.ma)

^d^Department of Pharmacognosy, Faculty of Pharmacy, MSA University, Giza 12585, Egypt.

mahelmy@msa.edu.eg

**^1^** Those authors contributed equally to the work.

*Corresponding author: [inas.younis@pharma.cu.edu.eg](mailto:inas.younis@pharma.cu.edu.eg) and [mansour.sobeh@um6p.ma](mailto:mansour.sobeh@um6p.ma)

**Supp. Table S1:** Origin of avocado seed extracts used in the study.

**Supp. Table S2:** *α*- amylase and *α*- glucosidase inhibition activity (µg/ml) of avocado seed extracts and acarbose.

**Supp. Fig.S1** BPC chromatograms of all avocado seeds extracts in the negative mode.

**Supp. Table S1:** Origin of avocado seed extracts used in the study.

| Sample weight | Identification Number | Sample Origin | Sample Code | Name of avocado |
| --- | --- | --- | --- | --- |
| 7.5±0.2 | 12-07-2016 I | Egypt | EH | **Hass** |
| 10.03±1.01 | 12-07-2016 II | Egypt | RE | **Reed** |
| 27.66±0.66 | 12-07-2016 III | South Africa | SA | **Pinkerton** |
| 12.53 ±0.15 | 12-07-2016 IV | Lebanon | LH | **Hass** |
| 13.86±0.35 | 12-07-2016 V | USA | UH | **Hass** |
| 8.53±0.25 | 24-09-2016 VI | Morocco | MH | **Hass** |
| 15.8±0.75 | 12-07-2016 VII | Kenya | KG | **Gwen** |

**Supp. Table S2:** *α*- amylase and *α*- glucosidase inhibition activity (µg/ml) of avocado seed extracts and acarbose

| Samples | α-amylase | α-glucosidase |
| --- | --- | --- |
| SA | 385.06 ± 2.97* | 115.40 ± 4.94* |
| LH | 990.37 ± 1.89* | 415.80 ± 4.03* |
| UH | 421.42 ± 2.66* | 123.80 ± 4.29* |
| EH | 110.08 ± 3.10* | 63.60 ± 2.56* |
| MH | 880.90 ± 3.63* | 412.00 ± 2.58* |
| RE | >1000* | 853.70 ± 4.51* |
| KG | 95.45 ±2.02* | 55.50 ± 2.48* |
| Acarbose | 34.71 ± 2.63 | 30.57 ± 0.58 |

*Significantly different from the positive control ( acarbose) at *p* ≤ 0.05

**Supp. Fig.S1** BPC chromatograms of all avocado seeds extracts in the negative mode.


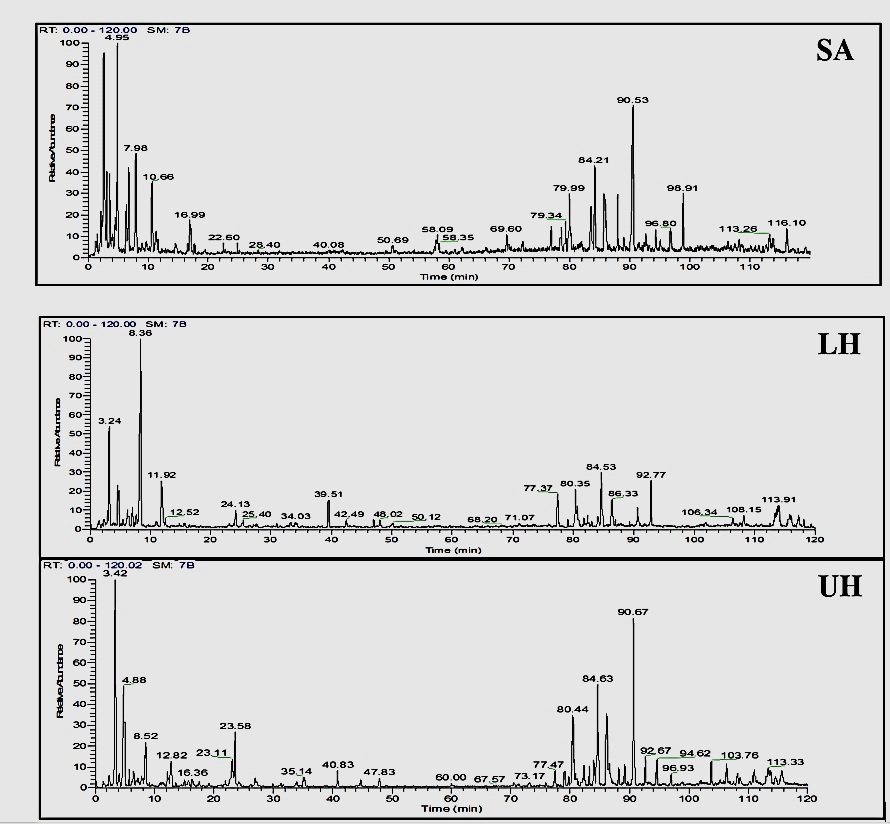


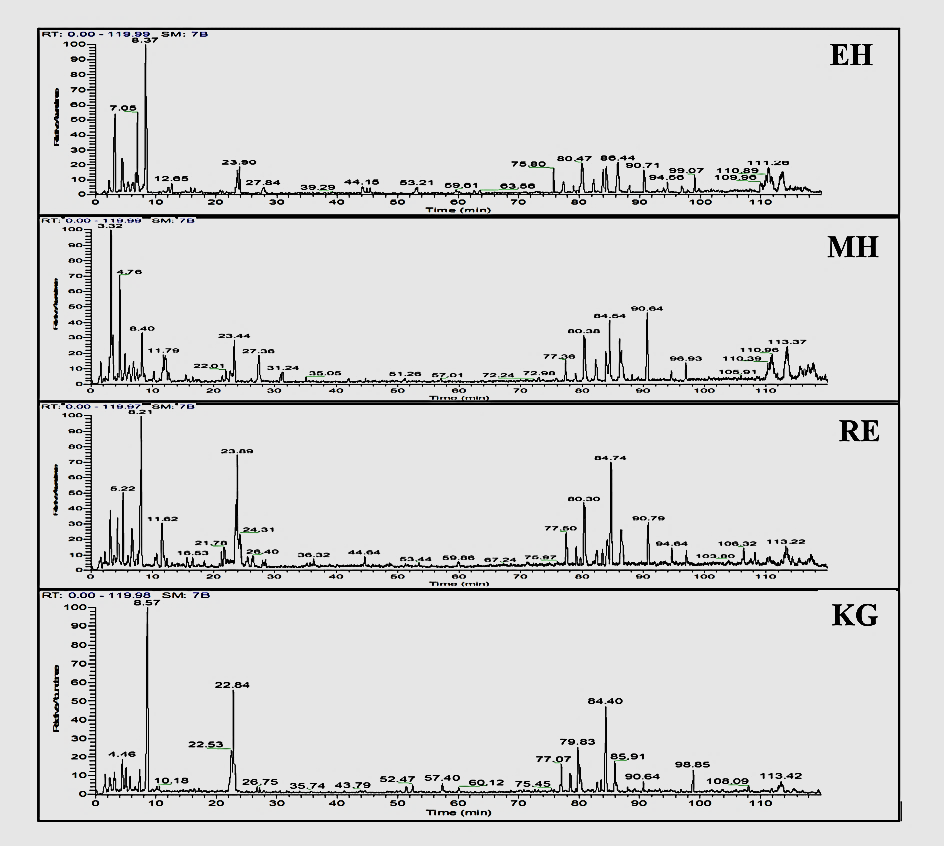

Supplement: Supplementary file 1 — Supplementary Information. [file 41598_2022_8479_MOESM1_ESM.docx]
